# Assessment of Alamandine in Pulmonary Fibrosis and Respiratory Mechanics in Rodents

**DOI:** 10.1155/2021/9975315

**Published:** 2021-05-18

**Authors:** Renata Streck Fernandes, Henrique Bregolin Dias, Wynnie Amaral de Souza Jaques, Tiago Becker, Katya Rigatto

**Affiliations:** ^1^Laboratório de Fisiologia Translacional, Universidade Federal de Ciências da Saúde de Porto Alegre (UFCSPA), Porto Alegre, Brazil; ^2^Programa de Pós-graduação em Ciências da Saúde, Universidade Federal de Ciências da Saúde de Porto Alegre (UFCSPA), Porto Alegre, Brazil; ^3^Laboratório de Biofísica Celular e Inflamação, Pontifícia Universidade Católica do Rio Grande do Sul (PUCRS), Porto Alegre, Brazil; ^4^Departamento de Engenharia Mecânica, Universidade Federal do Rio Grande do Sul, Porto Alegre, Brazil

## Abstract

**Introduction:**

Pulmonary fibrosis (PF) is characterized by an accelerated decline in pulmonary function and has limited treatment options. Alamandine (ALA) is a recently described protective peptide of the renin-angiotensin system (RAS) with essential tasks in several conditions. Our group previously demonstrated that ALA is reduced by 365% in the plasma of patients with idiopathic PF, and thus, it is plausible to believe that stimulation of this peptide could represent an important therapeutic target. In this sense, this study investigates the effects of ALA in an experimental model of PF.

**Materials and Methods:**

Bleomycin (BLM) was administrated in Wistar rats, and these fibrotic animals were treated with ALA for 14 days. Body weight, histology, respiratory, and hemodynamic parameters were analyzed to study the effects of ALA.

**Results:**

ALA treatment attenuated the development of fibrosis (*P* < 0.0001), reduced respiratory system elastance (*P* < 0.0001), and preserved weight gain (*P* < 0.0001) in fibrotic animals without affecting the autonomic control of blood pressure and heart rate.

**Conclusion:**

The data from this study demonstrate the potential of ALA to alleviate pulmonary fibrosis and improve respiratory system mechanics *in vivo*. The promising results encourage more detailed investigations of the potential of ALA as a future and efficient antifibrotic.

## 1. Introduction

Pulmonary fibrosis (PF) is characterized by the excessive extracellular matrix (ECM) deposition and frequently evolve to death [1]. In these patients, the elastance of the respiratory system are significantly increased [2], requiring greater respiratory work. Therefore, it is essential to find effective therapeutic strategies that facilitate lung expansion, reducing episodes of respiratory failure.

The participation of renin-angiotensin system (RAS) in PF has been well described [3–5]. The angiotensin-converting enzyme 2 (ACE2) has shown been an important counter-regulatory axis in several different conditions [6–8]. The alamandine (ALA), generated by ACE2 axis, although recently discovered [9], is well-known due its protective action in the cardiovascular system, i.e., vasodilation [9] and antifibrotic effects [10]. In addition, our previous study showed that patients with idiopathic PF present 365% less ALA in plasma [11], which probably indicates that the exogenous administration of this peptide may attenuate the development of PF and, consequently, reduce the decline in lung function.

Therefore, since bleomycin (BLM) is the best characterized PF model [12, 13], this study is aimed at evaluating the protective effect of ALA on the development of PF in BLM-induced rats.

## 2. Materials and Methods

### 2.1. Animals

All procedures followed the Guide for the Care and Use of Laboratory Animals published by the US National Institute of Health [14]. Ethics Research Committee of the Universidade Federal de Ciências da Saúde de Porto Alegre approved the study (protocol 17/207). Five-week-old male Wistar rats were housed in a room (25 ± 2°C) on a 12 : 12 h dark/light circadian rhythm with access to standard diet and water *ad libitum*.

### 2.2. Pulmonary Fibrosis Protocol

Rats were anesthetized with ketamine (80 mg/kg) and xylazine (10 mg/kg), and BLM (2.5 mg/kg, Bonar, Ache) or saline (0.9%) was administered by oropharyngeal aspiration (OA). On the same day, the miniosmotic pumps (OM; Alzet 2004) containing saline or ALA (Sigma Aldrich, St. Louis, MO, USA) solution were introduced subcutaneously onto the animal's back to deliver 0.25 *μ*l/hour (ALA; 50 *μ*g/kg or saline = 0.9% a day) for 14 days, respectively. The rats' health status and body weight were monitored daily.

Groups (*N* = 7/per group) are as follows: (1) CO: saline by OA and OM. (2) ALA: saline by OA and ALA (50 *μ*g/kg/day) in the OM. (3) BLM: BLM (2.5 mg/kg) by OA and saline in the OM. (4) BLM+ALA: BLM (2.5 mg/kg) by OA and ALA (50 *μ*g/kg/day) in the OM.

### 2.3. Histopathology

The lung was inflated and fixed in 10% phosphate-buffered formalin, and 5 *μ*m thickness sections were stained with hematoxylin and eosin (HE) and Masson's trichrome (TM). 20 fields of each slide were examined at a magnification of 400x. The fibrosis classification was according to the modified Ashcroft score [15]. Quantitative analysis of stained collagen area was performed using the Image Pro-Plus® 6.0 software (Media Cybernetics, Inc., Rockville, MD, USA).

### 2.4. Assessment of Respiratory Function

On day 14, animals were anesthetized with ketamine (80 mg/kg) and xylazine (10 mg/kg), positioned on a plane surgical table and tracheostomized to introduce a rigid-type cannula (2-mm ID). The cannula was fixed to the trachea by a silk thread and connected to the mechanical ventilator. Respiratory function was analyzed using a mechanical ventilator for small animals (FlexiVent, Scireq, Montréal, Canada) [16].

### 2.5. Hemodynamic Analyses and Autonomic Evaluation

After respiratory mechanics data collection, still under anesthesia, a polyethylene catheter (PE-50) was inserted into the right carotid to record arterial blood pressure (ABP) for 10 minutes (sample rate = 2000 Hz/channel). The analogical signals were digitalized by a data acquisition system (Windaq-AT/CODAS, Dataq 143 Instruments Inc., OH, USA). The data were analyzed by spectral analysis to assess the sympathovagal balance in the cardiovascular system [17, 18]. At the end of the experiment, animals were euthanized by intramuscular anasthetic overdose (240 mg/kg of ketamine and 30 mg/kg of xylazine) for lung collection.

### 2.6. Statistical Analysis

The normal distribution was tested by the Shapiro-Wilk test. Parametric data analysis was performed using one-way or two-way analysis of variance (ANOVA) followed by Tukey's multiple comparison test. For data with nonnormal distribution, the Kruskal-Wallis test was used, followed by the Dunn *post hoc* test. The associations between data were demonstrated through Pearson's correlation test. Data analysis was performed using the GraphPad Prism 8 software and presented as mean ± SEM. *P* < 0.05 was considered statistically significant.

## 3. Results

### 3.1. Alamandine Attenuates Loss Weight of Pulmonary Fibrosis

**Figure 1:**
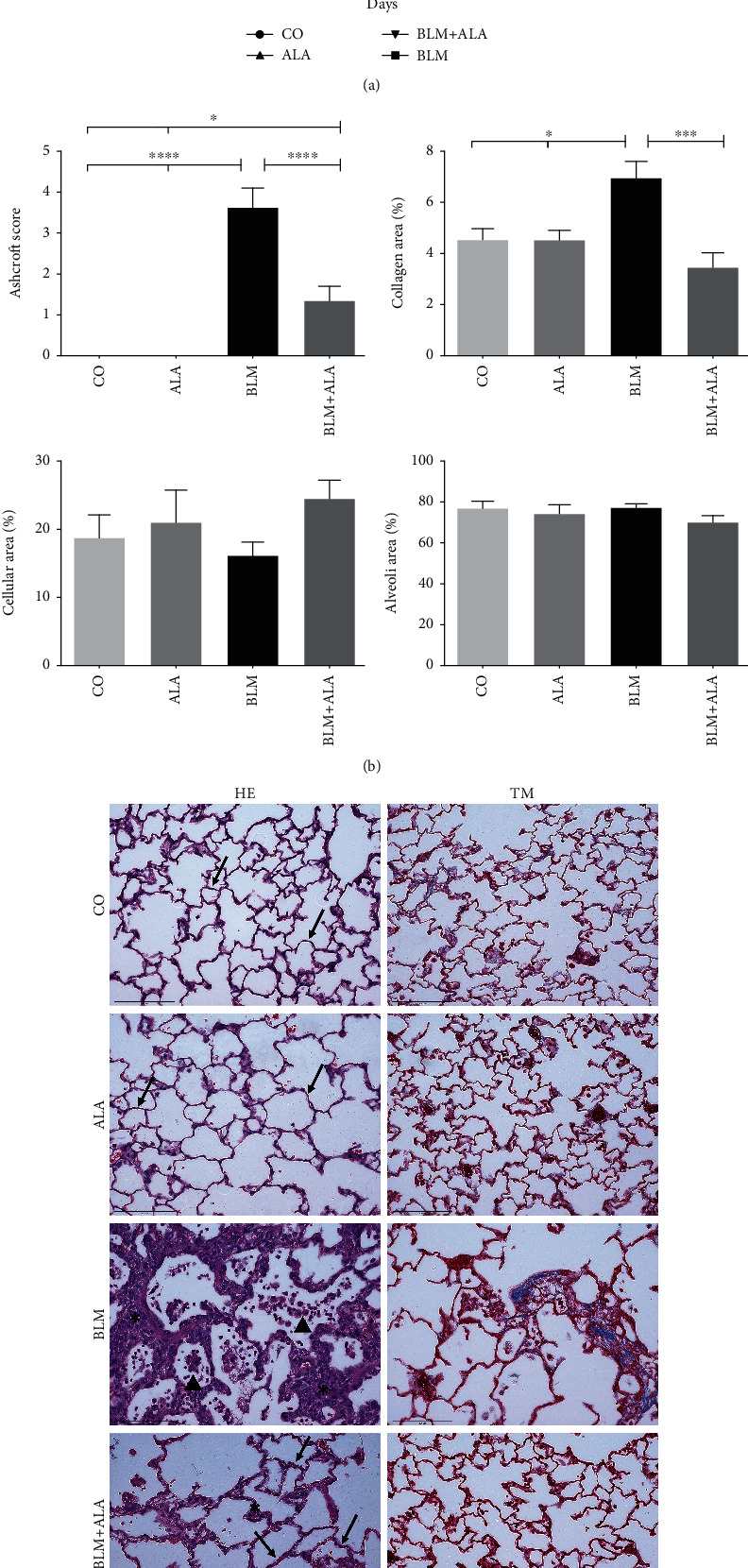
Effect of alamandine on development of pulmonary fibrosis. (a) Bodyweight variation (%) analyzed by two-way analysis of variance (ANOVA) followed by Tukey's multiple comparison posttest. (b) Ashcroft modified score (*F* = 33.10) and collagen area quantification in the lungs (*F* = 8.285). (c) Representative images of effects at two weeks after alamandine treatment on histological findings and collagen deposition in the lungs. Hematoxylin and eosin (HE) and Masson's trichrome (TM) staining. Magnification at 400x. CO: animals that received only saline; ALA: saline intratracheally and alamandine in the osmotic minipumps; BLM: bleomycin intratracheally and saline in the osmotic minipumps; BLM+ALA: bleomycin intratracheally and alamandine in the osmotic minipumps. Arrows: alveolar septa; asterisks: fibrous bands or fibrous masses; arrowhead: inflammatory cells. Tissue changes were analyzed by one-way ANOVA, followed by Tukey's multiple comparison posttest. All data represent mean ± SEM; *n* = 7 − 9. *P* < 0.05 was considered statistically significant. ^∗^*P* < 0.05; ^∗∗∗^*P* < 0.001; ^∗∗∗∗^*P* < 0.0001.

From day 6, the BLM group gained less weight onwards when compared to the CO and ALA groups (*P* < 0.01). ALA treatment had a protective effect starting on day 8 (*P* < 0.05). By day 14, PF rats gained significantly less body weight compared to the CO and ALA groups (*P* < 0.0001). However, animals treated with ALA maintained a similar weight to healthy animals (*P* < 0.0001, vs. BLM group; Figure 1(a)).

### 3.2. Alamandine Attenuates the Development of Pulmonary Fibrosis

The lung parenchyma was preserved in the CO and ALA groups (score = 0) compared to the BLM group (score = 3.6). ALA treatment had a potent effect (Figure 1(b)) by attenuating fibrosis (BLM + ALA score = 1.3; *P* < 0.0001; *F* = 33.10). Moreover, collagen deposition was confined to the regions around blood vessels and airways in the CO and ALA groups. As expected, there was considerable lung interstitial collagen deposition in the BLM group (*P* < 0.05 vs. CO and ALA). ALA treatment reduced significantly the collagen deposition compared to the BLM group (*P* < 0.001; *F* = 8.285). Cellular and alveolar areas were similar among groups in (Figure 1(b)). Images of lung histology are shown in Figure 1(c).

### 3.3. Alamandine Treatment Improves the Respiratory Mechanics in Pulmonary Fibrosis

**Figure 2:**
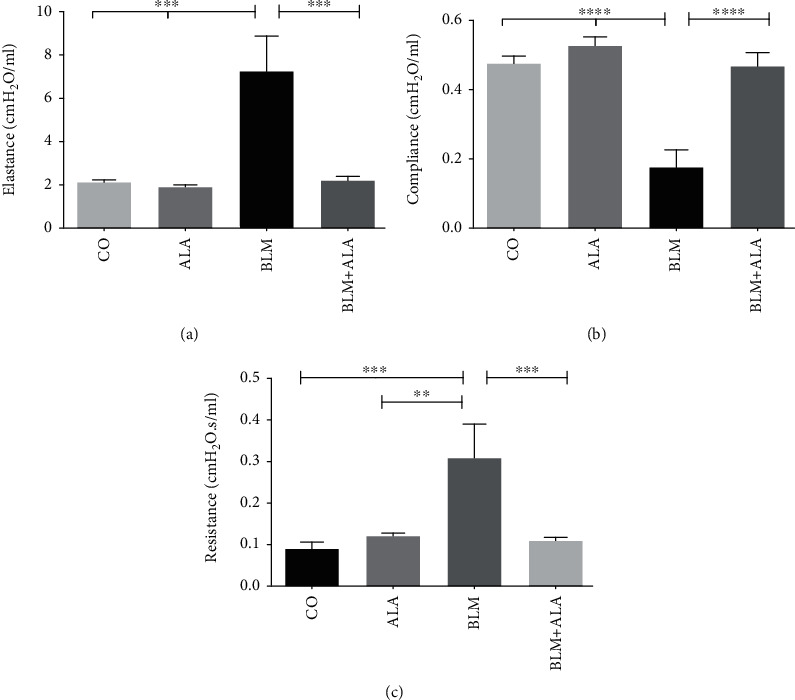
Lung mechanics on day 14. (a) Elastance, *F* = 11.28. (b) Compliance, *F* = 20.50. (c) Resistance: level of constriction, *F* = 8.672. CO: animals that received only saline; ALA: saline intratracheally and alamandine in the osmotic minipumps; BLM: bleomycin intratracheally and saline in the osmotic minipumps; BLM+ALA: bleomycin intratracheally and alamandine in the osmotic minipumps. One-way ANOVA followed by Tukey's multiple comparison test was used. Data represent mean ± SEM; *n* = 6 − 7; *P* < 0.05 was considered statistically significant. ^∗∗^*P* < 0.01; ^∗∗∗^*P* < 0.001; ^∗∗∗∗^*P* < 0.0001.

There was significant increase in the dynamic elastance (Edyn) in the BLM group (7.27 ± 1.6 cmH_2_O/ml) compared to the CO (2.13 ± 0.10 cmH_2_O/ml) and ALA (1.92 ± 0.08 cmH_2_O/ml) groups (Figure 2(a)). The BLM+ALA group demonstrated a significant attenuation in Edyn (2.22 ± 0.18 cmH_2_O/ml) compared to the BLM group (*P* < 0.001; *F* = 11.28; Figure 2(a)).

As expected, the dynamic compliance was lower in the BLM group (0.18 ± 0.04 cmH_2_O/ml) vs. CO (0.48 ± 0.02 cmH_2_O/ml) and ALA (0.53 ± 0.02 cmH_2_O/ml) groups. The results from the BLM+ALA (0.47 ± 0.04 cmH_2_O/ml) demonstrated a protective role of ALA on respiratory mechanics (*P* < 0.0001 vs. BLM; *F* = 20.50; Figure 2(b)). Moreover, the BLM group showed significantly higher respiratory dynamic resistance (*P* < 0.001 vs. CO; *F* = 8.672; Figure 2(c)), indicating the protective effect ALA in the BLM+ALA group (*P* < 0.001) to prevent loss of tissue function.

**Figure 3:**
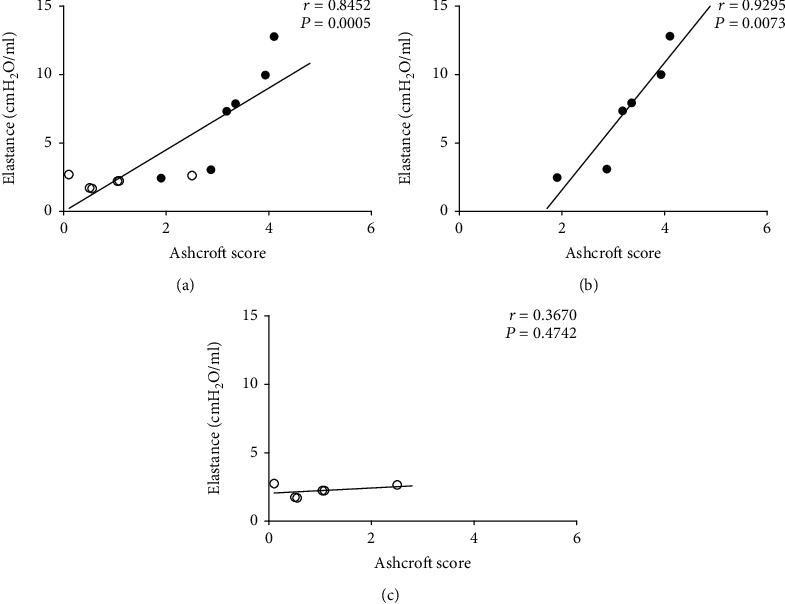
Pearson's correlation between Ashcroft score and pulmonary elastance. (a) BLM and BLM+ALA groups (*R*^2^ = 0.7143). (b) BLM group (*R*^2^ = 0.8640). (c) BLM+ALA group (*R*^2^ = 0.1347). BLM: bleomycin intratracheally and saline in the osmotic minipumps; BLM+ALA: bleomycin intratracheally and alamandine in the osmotic minipumps; *N* = 7. •: BLM group. °: BLM+ALA group.

The correlation between the Ashcroft score and pulmonary elastance is shown in Figure 3. There was a strong positive correlation between the BLM and BLM+ALA groups (*r* = 0.8452; *R*^2^ = 0.7143; *P* = 0.0005; Figure 3(a)) probably due to the data from the BLM group alone (*r* = 0.9295; *R*^2^ = 0.8640; *P* = 0.0073; Figure 3(b)). Observing the correlation between those parameters in the BLM+ALA group, it is possible to verify that this association was weak (*r* = 0.3670; *R*^2^ = 0.1347; *P* = 0.4742; Figure 3(c)).

### 3.4. Hemodynamic Analyses

Table 1 shows that there were no differences among the hemodynamic parameters. The ABP (*P* = 0.8121) and heart rate (*P* = 0.1432) were not different among groups. This result was confirmed by spectral analysis showing that the autonomic nervous system participation in the heart was also not different among groups. The sympathetic (LF) and parasympathetic components (HF) were similar in absolute and normalized units. These results were confirmed by the LF/HF ratio. In addition, the 0 V, which represents the sympathetic participation to the heart through symbolic analysis, also indicates that there was no significant difference among groups.

## 4. Discussion

We have demonstrated for the first time the protective effect of ALA in attenuating PF and preserving respiratory mechanics. In addition, BLM administration significantly induced weight loss, which was prevented by ALA treatment.

According to American Thoracic Society recommendations, induction by intratracheal BLM in male rats is the one that best mimics the disease in humans and is the most suitable for initial preclinical tests [19]. Kilic et al. [20] showed that 2.5 mg/kg of BLM in Wistar rats causes histological changes compatible with PF. Our study also demonstrated that this dose is sufficient to study the effects of antifibrotic substances without causing lethal damage to animals. In addition, George et al. [21]describes that studies for antifibrotic therapies using the BLM animal model of PF can be beneficial also to COVID-19. Even in patients recovered from COVID-19, the virus elimination does not preclude the development of progressive fibrosis. Thus, the promising results of ALA obtained in our study might encourage investigations of preventive fibrosis therapies after SARS-CoV-2 infection.

Our findings agree with the literature showing that PF leads to a decrease in body weight [22], a strong indication of health in animals [22]. While ALA treatment prevented the loss in body weight, the higher energy consumption to respiratory work probably explains this decrease in BLM group. This is true also for humans because treatment with nintedanib or pirfenidone normally provokes weight loss in patients [23], contributing to their poor prognosis [24].

Furthermore, the antifibrotic effect of ALA has already been described in the cardiovascular system [25] and, more recently, in the liver [26]. Although there are strong indications of the protective effects of ALA, there are no reports in the literature investigating its action on the fibrotic process in the lung. To date, there are only studies suggesting the protective role of to ACE2 and angiotensin-(1–7) in PF [27] and COVID-19 [8, 28]. In this study, ALA alleviated the lung degree of fibrosis and collagen deposition. It is established in the literature that collagen is the primary determinant of overall lung tissue elasticity [29], which therefore commits to functional capacity [30] in the same proportion as the degree of fibrosis [31]. Thus, if ALA prevent the fibrosis, it is possible that ALA can also act by improving respiratory mechanics.

Furthermore, our findings show that the degree of fibrosis was positively correlated with respiratory system elastance and that ALA treatment reduced this correlation. It is well established that fibrosis increases the elastic recoil forces of the lung and therefore reduces lung compliance. Moreover, excess ECM alters ventilation/perfusion ratios in the lung, causing hypoxemia both at rest and with effort [32]. Consequently, our results indicate that treatment with ALA might overcome these mechanical changes and could be effective in reducing respiratory work also in IPF patients. Despite evidence in the literature regarding the involvement of the RAS in PF [33], this is the first study which demonstrates the protective effect of ALA in lungs, extending the knowledge about the potential of the ACE2 axis [34]. In this sense, our data point to the possibility of using ALA to treat PF of varying etiology, mainly when involving ACE2 participation. As proposed by Wu [8], our findings indicate that the compensation of ACE2 function, with ALA administration, could be a promising alternative to treat the severe respiratory damage provoked by PF, as found in COVID-19 [21, 28].

Studies have also indicated that ALA has different effects on arterial blood pressure (ABP) regulation, depending on where it is administered and/or the pathophysiological condition. It causes an increase in ABP and sympathetic participation when injected into the paraventricular nucleus of spontaneously hypertensive rats [35], whereas subcutaneous infusion of ALA attenuates hypertension [36]. It was also demonstrated by Wang et al. [37] that ALA attenuates cardiac fibrosis caused by long-term hypertension independently of ABP. Similar to the report by Wang et al. in 2019 [37], our results demonstrate that ALA attenuated lung fibrosis but did not change ABP, clearly indicating the versatility of ALA's effects and confirming RAS pleiotropism.

## 5. Conclusion

In the future, it is possible that ALA represents an important strategy to improve IPF patient quality of life. This histological and functional study supports a significant progress and may encourage further investigation into the mechanisms of ALA in PF.

## Figures and Tables

**Table 1:** Hemodynamic data and spectral and symbolic analysis results. CO: control rats; ALA: rats treated only with alamandine; BLM: rats treated with bleomycin; BLM+ALA: rats treated with bleomycin+alamandine. HR: heart rate; bpm: beats per minute; ABP: average blood pressure. HRV: heart rate variability; LF: low- and HF: high-frequency component; a: absolute and nu: normalized units. A one-way analysis of variance (ANOVA) followed by Tukey's multiple comparison posttest was used for ABP and HR evaluation. The Kruskal-Wallis and the *post hoc* Dunn's multiple comparison tests were performed to detect differences in spectral and symbolic analysis. Data represent mean ± SEM, and a *P* < 0.05 was considered statistically significant.

Spectral analysis					
	CO (*n* = 7)	ALA (*n* = 7)	BLM (*n* = 7)	BLM+ALA (*n* = 7)	*P*
ABP (mmHg)	75 ± 6	70 ± 3	74 ± 5	77 ± 3	0.81
HR (bpm)	299 ± 16	269 ± 17	251 ± 11	267 ± 10	0.14
HRV (ms^2^)	9.18 ± 2.26	9.58 ± 2.81	9.43 ± 4.28	12.69 ± 6.68	0.90
LFa (ms^2^)	2.12 ± 0.74	3.15 ± 1.38	1.31 ± 0.37	2.12 ± 0.86	0.68
HFa (ms^2^)	5.50 ± 1.63	4.30 ± 0.98	7.07 ± 4.12	6.03 ± 2.47	0.96
LFnu	0.32 ± 0.09	0.32 ± 0.07	0.28 ± 0.08	0.30 ± 0.07	0.96
HFnu	0.68 ± 0.09	0.68 ± 0.07	0.72 ± 0.08	0.70 ± 0.07	0.96
LF/HF ratio	0.83 ± 0.38	0.62 ± 0.19	0.56 ± 0.21	0.50 ± 0.16	0.97
Symbolic analysis (%)					
0V pattern	0.101 ± 0.006	0.100 ± 0.019	0.111 ± 0.027	0.111 ± 0.010	0.99
1V pattern	0.370 ± 0.010	0.372 ± 0.021	0.361 ± 0.017	0.392 ± 0.010	0.40
2LV pattern	0.102 ± 0.014	0.091 ± 0.012	0.103 ± 0.012	0.075 ± 0.014	0.34
2UV pattern	0.418 ± 0.020	0.434 ± 0.031	0.424 ± 0.035	0.413 ± 0.020	0.94

## Data Availability

The experimental data that support the findings of this study are included within the article.
